# Exploring the Microbial Community Structure in the Chicken House Environment by Metagenomic Analysis

**DOI:** 10.3390/ani14010055

**Published:** 2023-12-22

**Authors:** Cheng Lou, Zhuo Chen, Yu Bai, Tongjie Chai, Yuling Guan, Bo Wu

**Affiliations:** 1 Guangdong Provincial Key Laboratory of Animal Molecular Design and Precise Breeding, School of Life Science and Engineering, Foshan University, Foshan 528225, China; lcwyzw@163.com (C.L.); 18736527586@163.com (Z.C.); baiyu4202@163.com (Y.B.); yulingguan1202@163.com (Y.G.); 2 College of Animal Science and Technology, Shandong Agricultural University, Tai’an 271000, China; chaitj117@163.com

**Keywords:** chicken house, PM2.5, aerosol, metagenomics, public health

## Abstract

**Simple Summary:**

Microorganisms suspended in the air are considered one of the main challenges leading to chicken respiratory diseases despite continuous production management and environmental prevention improvements. Traditional feeding modes have some disadvantages, with high density and poor ventilation; therefore, they provide good conditions for microorganisms to survive. The complex composition of microorganisms in the chicken house environment is unknown, especially regarding viruses. However, few works have paid attention to the microbial community structure and its potential risks to animal and human health.

**Abstract:**

The environmental conditions of chicken houses play an important role in the growth and development of these animals. The chicken house is an essential place for the formation of microbial aerosols. Microbial aerosol pollution and transmission can affect human and animal health. In this work, we continuously monitored fine particulate matter (PM2.5) in the chicken house environment for four weeks and studied the microbial community structure in the aerosols of the chicken house environment through metagenomic sequencing. Our results found that bacteria, fungi, viruses, and archaea were the main components of PM2.5 in the chicken house environment, accounting for 89.80%, 1.08%, 2.06%, and 0.49%, respectively. Conditional pathogens are a type of bacteria that poses significant harm to animals themselves and to farm workers. We screened ten common conditional pathogens and found that *Staphylococcus* had the highest relative abundance, while *Clostridium* contained the most microbial species, up to 456. *Basidiomycetes* and *Ascomycota* in fungi showed dramatic changes in relative abundance, and other indexes showed no significant difference. Virulence factors (VF) are also a class of molecules produced by pathogenic microbes that can cause host diseases. The top five virulence factors were found in four groups: FbpABC, HitABC, colibactin, acinetobactin, and capsule, many of which are used for the iron uptake system. In the PM2.5 samples, eight avian viruses were the most significant discoveries, namely Fowl aviadovirus E, Fowl aviadovirus D, Avian leukosis virus, Avian endogenous retrovirus EAV-HP, Avian dependent parvovirus 1, Fowl adenovus, Fowl aviadovirus B, and Avian sarcoma virus. The above results significantly improve our understanding of the microbial composition of PM2.5 in chicken houses, filling a gap on virus composition; they also indicate a potential threat to poultry and to human health. This work provides an important theoretical basis for animal house environmental monitoring and protection.

## 1. Introduction

Microbial aerosols are a significant cause of air pollution and disease transmission [1,2,3]. Microbial aerosols form when microorganisms are suspended together with dry solid or liquid particles in the air [4]. Microbial aerosols contain complex components, and they can be divided into bacterial aerosols, fungal aerosols, and viral aerosols according to their principal features [5,6]. Compared with other animal houses, the concentrations and components of microbial aerosols in chicken houses are the highest and the most abundant, respectively [7]. During intensive poultry breeding, chicken houses are also prone to high concentrations of microbial aerosols, increasing the risk of respiratory diseases and severely reducing animal production performance [8,9]. In particular, PM2.5 from poultry houses has adverse effects on the health of animals and workers [10].

PM2.5, including particles with a mean aerodynamic diameter below 2.5 µm, is small in size and inhaled to a significant depth, which can cause harmful health effects on the pulmonary system [11,12,13]. The concentration and respiratory risks from PM2.5 in chicken houses are higher than in other kinds of animal houses [14]. Our previous research showed that long-term exposure to high levels of PM2.5 significantly reduced the growth performance of poultry, pulmonary damage, and metabolome alterations [15]. Similar results were seen in research about the correlations between hospital admissions and PM2.5. The latest research results have found a significant correlation between PM2.5 and hospitalization rates for pneumonia and asthma in children under five years old. For every 10-unit increase in lag03 air pollutant concentration, hospitalization of children with pneumonia and asthma increased by 2.22% [16]. In addition, PM2.5 is susceptible to including toxic and harmful substances (e.g., heavy metals, microorganisms, etc.), which may enter the circulatory system or trigger an inflammatory response in the lungs after entering the organism, which in turn hurts the cardiovascular system [17,18]. In areas with low levels of urbanization, particulate matter (PM) is closely related to the daily peak of cardiovascular admissions. For every 10 μg/m^3^ increase in PM2.5, the number of people admitted to the hospital due to cardiovascular disease increased by 0.97% [19]. Long-term exposure to PM2.5 not only has pathogenicity to animals raised in an animal house but also poses a threat to farm workers and surrounding residents, resulting in a broader range of health risk effects. Therefore, exploring the distribution characteristics and concentrations of microbial aerosols and PM2.5 in chicken houses is necessary, which will provide a theoretical basis for understanding the correlation between PM2.5 in the chicken house environment and human and animal health.

Initial efforts to characterize the microbiome in PM2.5 from animal houses involved using 16S rRNA sequencing [20,21], which has several limitations. Metagenome-sequencing-based surveys of environmental microbiomes can provide more accurate and descriptive insights into taxonomic diversity and functional potential. Xu et al. analyzed the community characteristics of the intestinal microbiome of Chinese native yellow-feathered broilers through metagenomic sequencing. The dominant phyla of yellow-feathered broilers were *Bacteroides* and *Firmicutes*. At the same time, among all types of antibiotic resistance found, tetracyclines, multidrugs, and aminoglycosides accounted for the majority [22]. Metagenomic sequencing is also a helpful method to analyze poultry diseases. Various pathogens cause avian respiratory disease complex (RDC). Researchers applied metagenomics to study its pathogenic virus groups and found nine virus families, of which RNA viruses contributed the most to RDC [23]. As an RNA virus, the negative impact of avian influenza virus on animal and human health is unquestionable. However, certain avian influenza viruses threaten humans more than poultry, such as H7N9. This virus has a low pathogenicity in poultry, but can cause severe infections in humans who come into contact directly with infected poultry. The most severe symptoms are severe lung disease and acute respiratory distress syndrome [24]. It follows that metagenomic sequencing is beneficial for analyzing the microorganisms in the animal house environment.

Microbial aerosols are an important indicator of air quality, which can have an impact on human and animal health. Therefore, this study aims to evaluate the composition and distribution characteristics of microbial aerosols in a chicken house to determine air quality. In this study, a semi-open chicken house in Qingyuan City, Guangdong Province, was used as an experimental site to randomly collect air samples for one month during the growth stage of Qingyuan partridge chickens, and the concentration and distribution characteristics of microbial aerosols in the chicken house were analyzed using high-throughput sequencing technology. This information will help us to understand the occurrence and spread of pathogens in chicken houses to better ensure the healthy growth of chickens. More importantly, it can enhance workers’ awareness of protection and reduce health risks in occupational environments like animal houses.

## 2. Materials and Methods

### 2.1. Sampling of PM2.5 and DNA Extraction

PM2.5 was collected using an air particulate sampler at a chicken farm (GPS at 113.016; 23.7) in Qingyuan City. The chicken farm in our study had 3000 broilers and allowed broilers to feed and drink freely. During the research process, we collected samples for four consecutive weeks, with three samples repeated every week to monitor the temporal changes in microbial aerosols in the chicken farm. PM2.5 was stored in a refrigerator at −80 °C for long-term storage. Temperature and humidity in the house were also recorded.

Total genomic DNA from the PM2.5 samples was extracted using the CTAB (Cetyl trimethylammonium bromide) method [25], and the concentration and the integrity of the collected DNA were determined using gel electrophoresis (1 percent agarose gel). After passing the quality inspection, the VAHTS Universal Plus DNA Library Pren Kit for Illumina was used to construct the sample library. The library was subjected to fragment quality inspection with Qsep-400, and the library concentration was quantified by Qubit 3.0. Illumina NovaSeq 6000 (Illumina, San Diego, CA, USA) was used for computer sequencing of the constructed library.

### 2.2. Metagenomic Sequencing

The raw sequences (raw reads) obtained by sequencing contained low-quality sequences. To ensure the quality of the information analysis, we filtered raw reads to get clean reads using Trimmomatic (Version 0.39) software. After receiving clean tags, host contamination was removed through Bowtie2. The MEGAHIT (Version 1.2.9) software was used to assemble the clean reads [26]. The results of the assembly were generally affected by factors such as the amount of sample sequencing data, species diversity, and uneven species abundance. The assembly results were evaluated for metagenomic component analysis. MetaGeneMark (version 3.26) was used to predict genes from assembled contigs [27]. The predicted genes were built into non-redundant gene sets using MMseqs2 [28], with a sequence similarity threshold of 95% and an alignment coverage threshold of 90% [29]. Based on non-redundant gene sets, we performed functional annotation and taxonomic analysis of general and unique databases and measured species composition and abundance information for the samples.

### 2.3. Taxonomic Assignment and Functional Annotation

The Nr database is a non-redundant protein database. It contains comprehensive protein sequence and annotation information, and corresponding species information is included in the annotation information. Taxonomic assignment of genes was performed with Blastp by aligning them against the Nr database to find the most similar sequence. The species composition and relative abundance information for the PM2.5 samples was obtained according to the species information of the most similar Nr sequences aligned with non-redundant genes. Then, Python was used to draw and display the species histogram at the levels of boundary, phylum, genus, and species taxonomy. From the graph, we can intuitively see the species composition of each sample and the proportion of different species in each sample.

### 2.4. Gene Function Analysis

We used public databases to predict the functions of predicted genes, including KEGG [30], CAZy [31], VFDB [32], and Nr (non-redundant protein sequence database) [33], with e-values lower than 10^−5^, and retrieved the proteins with the highest sequence similarity with given genes along with their functional protein annotations.

### 2.5. Statistical Analysis

For diversity analysis, overall differences in the microbial community structures were investigated using principal coordinate analysis (PCoA) based on the abundance table for each taxonomic hierarchy using R language tools. PCoA was based on the Bray–Curtis distance value abundance table for each taxonomic order. LEfSe analysis was used to look for differences among the groups. The significance of differences among groups was checked by the Kruskal–Wallis test, and values of *p* < 0.05 were regarded as significant.

## 3. Results and Discussion

### 3.1. Sequencing, Assembly, and Microbial Taxonomy

Metagenomic analysis of PM2.5 DNA was performed using the Illumina HiSeq platform to assess the diversity of the microbial community and potential proteins/enzymes involved in the biomass process. A total of 94,268.89 Mbp of raw data and an average of 7855.74 Mbp per sample were generated from all processed models. After cleaning raw reads, an average of 7774.87 Mbp of clean data per sample remained (Supplementary Table S1).

**Figure 1:**
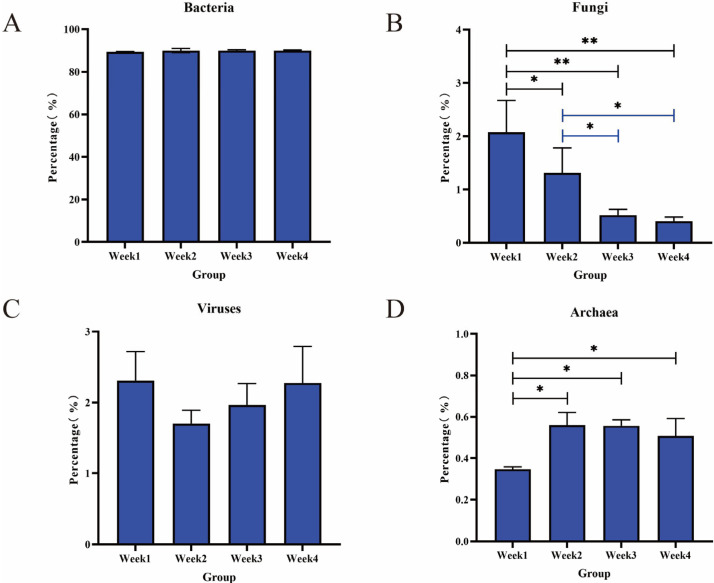
Relative abundance of bacteria (**A**), fungi (**B**), viruses (**C**), and archaea (**D**) in the PM2.5 samples for four weeks. * *p* < 0.05, ** *p* < 0.01.

The genes obtained from the metagenomic analysis of the PM2.5 samples were annotated in the Nr database. Of these, approximately 89% were assigned to bacteria, followed by viruses and fungi, with archaea at a low abundance (less than 0.5%, Figure 1). Overall, 2.06% and 1.08% of PM2.5 DNA were obtained from viruses and fungi, respectively. Despite the similarities in microbial community structures in all samples, fungi and archaea abundance changes were visible. The relative abundance of fungi during four weeks tended to decrease (*p* < 0.05) (Figure 1B). In the fourth week, fungi had the lowest abundance (0.41%). Meanwhile, archaea abundance in the first week was significantly lower than in other weeks (*p* < 0.01) (Figure 1D). We speculate that there may be a certain antagonistic relationship between fungi and archaea. To further identify the microbial community’s composition, we emphasized analyzing the species, especially bacteria, fungi, viruses, and archaea, at different taxonomic levels (from the phylum level to the genus level).

### 3.2. Bacterial Communities and Diversity Analysis

**Figure 2:**
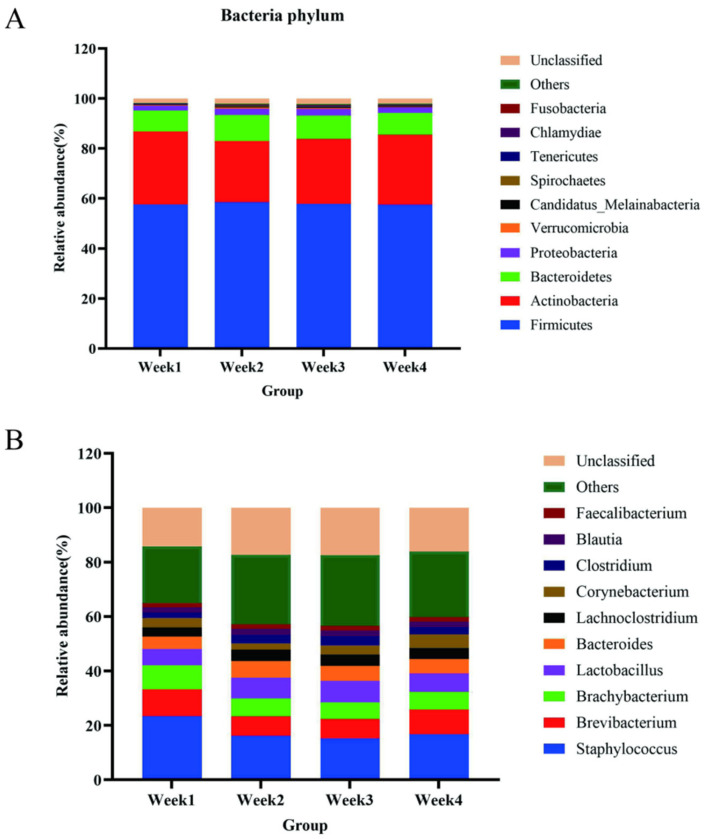
The top 10 most abundant bacterial phyla (**A**) and genera (**B**) in the PM2.5 samples for four weeks.

In our study, 145 bacterial phyla and 2377 bacterial genera were detected in the PM2.5 samples (Supplementary Table S2). One-way ANOVA analysis of the alpha diversity index showed a significant difference in the indices of Shannon, Simpson, ACE, and Chao1, suggesting that the community richness and evenness had changed during sampling (*p* < 0.05, Supplementary Figure S1). The relative abundances of the bacterial communities at the phylum and genus levels for each sample are shown in Figure 2. *Firmicutes* had the highest quantity (57.81%, Figure 2A); thus, it plays a vital role in the air environment and even leads to severe diseases. *Firmicutes* are one of the major microbial phyla in nature. At the same time, *Firmicutes* are also one of the main components of intestinal microorganisms in poultry [34]. A variety of microorganisms within the phylum *Firmicutes* have been found to be capable of causing damage to poultry, such as *Staphylococcus aureus*, *Streptococcus*, *Clostridium botulinum*, *Clostridium perfringens*, and so on [35,36,37]. In addition, *Actinobacteria, Bacteroidetes, Proteobacteria*, and *Verrucomicrobia* accounted for 26.91%, 9.30%, 2.30%, and 0.34% of bacteria, respectively. Therefore, the five dominating phyla accounted for 96.66% of bacteria, and the number of *Firmicutes* was 6.22-fold higher than that of *Bacteroidetes*.

**Figure 3:**
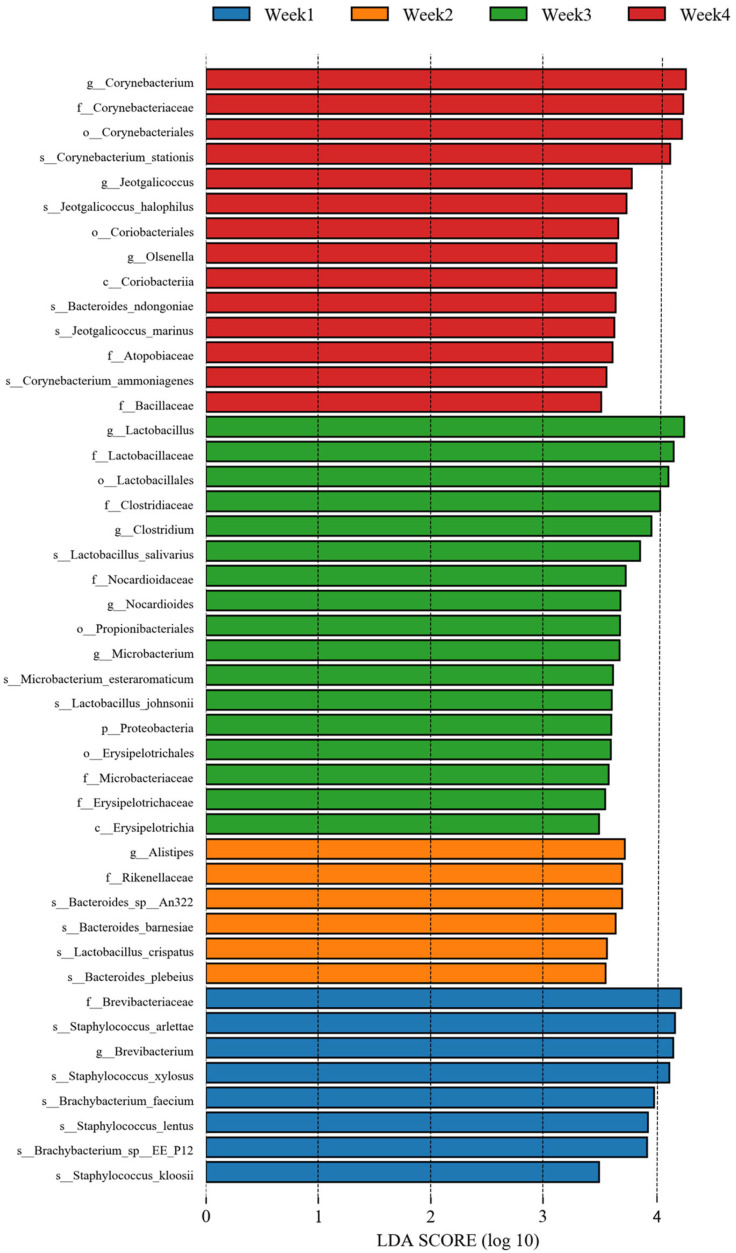
LDA distribution histogram of bacteria for four weeks identified by the LEfSe algorithm. Only taxonomic biomarkers (LDA score > 3.5) are presented.

Similarly, analysis at the genus level showed that 2377 bacteria were identified (Supplementary Table S2), with *Staphylococcus*, *Brevibacterium*, *Brachybacterium*, *Lactobacillus*, *Bacteroides*, *Lachnoclostridium*, *Corynebacterium*, *Clostridium*, *Blautia*, and *Faecalibacterium* as the top 10 genera. *Staphylococcus* (17.83%), belonging to *Firmicutes*, was dominant, followed by *Brevibacterium* (8.33%) and *Brachybacterium* (7.04%), belonging to *Actinobacteria* (Figure 2B). We also conducted LEfSe and linear discriminant analysis (LDA) using the relative abundances of the genera to find the differential bacterial taxa among the groups. Biomarkers found in all groups included *Corynebacterium*, *Brevibacterium*, and *Lactobacillus* (LDA score > 4.0) (Figure 3).

Regarding Beta diversity, PCoA was used to explore the similarity of bacterial community distribution characteristics in different groups (Supplementary Figure S2). The results showed that the samples of different groups separated, indicating that all groups’ bacterial communities were quite different (*p* < 0.05). The age of the chickens affects the colonization of intestinal microorganisms, so it may also be an essential factor affecting microorganisms in microbial aerosols [38,39]. In a word, it is meaningful to analyze microbial aerosols by grouping chickens into different age groups.

### 3.3. Opportunistic Pathogens Were Discovered in the PM2.5 Samples

Opportunistic pathogens, one of the primary pollution sources, have attracted much attention. We evaluated the pollution of opportunistic pathogens at a genus level, mainly investigating the distribution of 10 kinds of opportunistic pathogens in the chicken house environment. As shown in Table 1, *Staphylococcus* was the most abundant (15.999%), followed by *Clostridium*, accounting for 2.486% of opportunistic pathogens. See Table 1 for the number of species detected in each genus mentioned above. The common conditional pathogens in the top 100 included *Staphylococcus aureus, Escherichia coli*, and *Staphylococcus epidermidis*.

Among the top ten genera with relative levels of abundance of bacteria obtained in this study, *Staphylococcus* had a high relative abundance. It can produce a variety of enterotoxins and other toxins and trigger inflammatory reactions by activating inflammatory cells. Many kinds of inflammation have been found to be related to *Staphylococcus*, such as keratitis, mastitis, etc. [40,41]. *Corynebacterium* can cause some organs and tissues to undergo septic or caseous pathological changes [42]; its pathogenic effect on birds was reported by Huang et al. [43]. A high abundance of opportunistic pathogens is a significant cause of infection and disease in many animal hosts, leading to a significant impact on public health and agriculture.

### 3.4. Fungal Communities and Diversity Analysis

**Figure 4:**
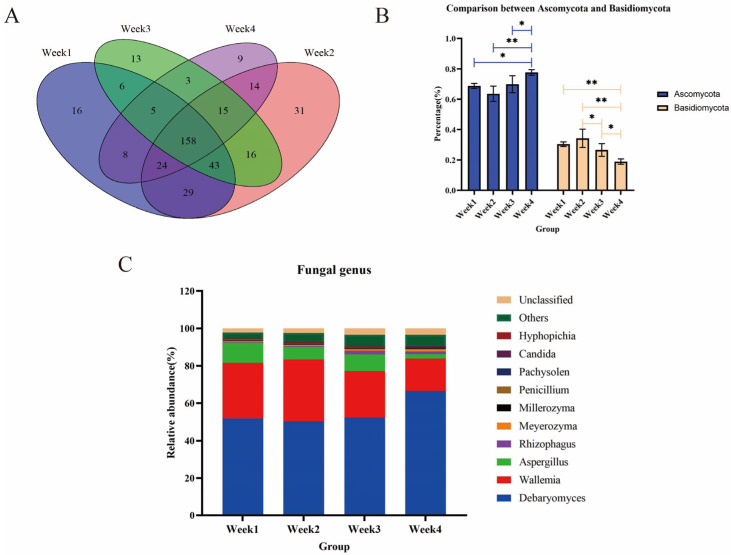
(**A**) Fungal genera sampling over four weeks is shown by the Venn diagram. (**B**) Comparative results for the relative abundance of *Ascomycota* and *Basidiomycota* in sampling over four weeks. (**C**) The top 10 most abundant fungal genera in the PM2.5 samples with sampling over four weeks. * *p* < 0.05, ** *p* < 0.01.

We analyzed the composition and structure of the fungal flora in the PM2.5 samples. A Venn diagram illustrates that the number of shared fungi in four groups was 158 at the genus level (Figure 4A). For unigenes assigned as fungi, samples in different periods had similar dominant fungal communities at the phylum level, including *Ascomycota* and *Basidiomycota*, representing more than 95% of fungi. Differences in relative abundance were observed between *Ascomycota* and *Basidiomycota* in all groups of samples (Figure 4B). The abundance of *Ascomycota* in the fourth week was higher than in other weeks (*p* < 0.05), but *Basidiomycota* had the opposite status (*p* < 0.05). The top five fungal genera at the genus level were *Debaryomyces, Wallemia, Aspergillus, Rhizophagus*, and *Meyerozyma* (Figure 4C). *Debaryomyces* (55.26%) was dominant, followed by *Wallemia* (26.27%) and *Aspergillus* (7.24%). In addition to the Simpson index, there were no significant differences in the Chao1, ACE, or Shannon indices in any group (Supplementary Table S3).

Fungi are also associated with diseases. For example, recent studies have found that *Debaryomyces* accumulated in the intestines of patients with Crohn’s disease (localized enteritis) and affected mucosal healing [44]. Therefore, fungi’s most significant harm may lie in it delaying wound healing, facilitating more microorganisms to invade the body, and affecting human and animal health. *Aspergillus* is a kind of opportunistic pathogen widely distributed in nature. It is an essential cause of mildew for many substances and can also cause primary invasive lung infection and spread to other organs [45]. The relative abundance of fungi was not high from the perspective of the entire microbial community. However, the pathogenic risk may increase if preventive measures are not taken.

### 3.5. Diversity and Abundance of Viruses

**Figure 5:**
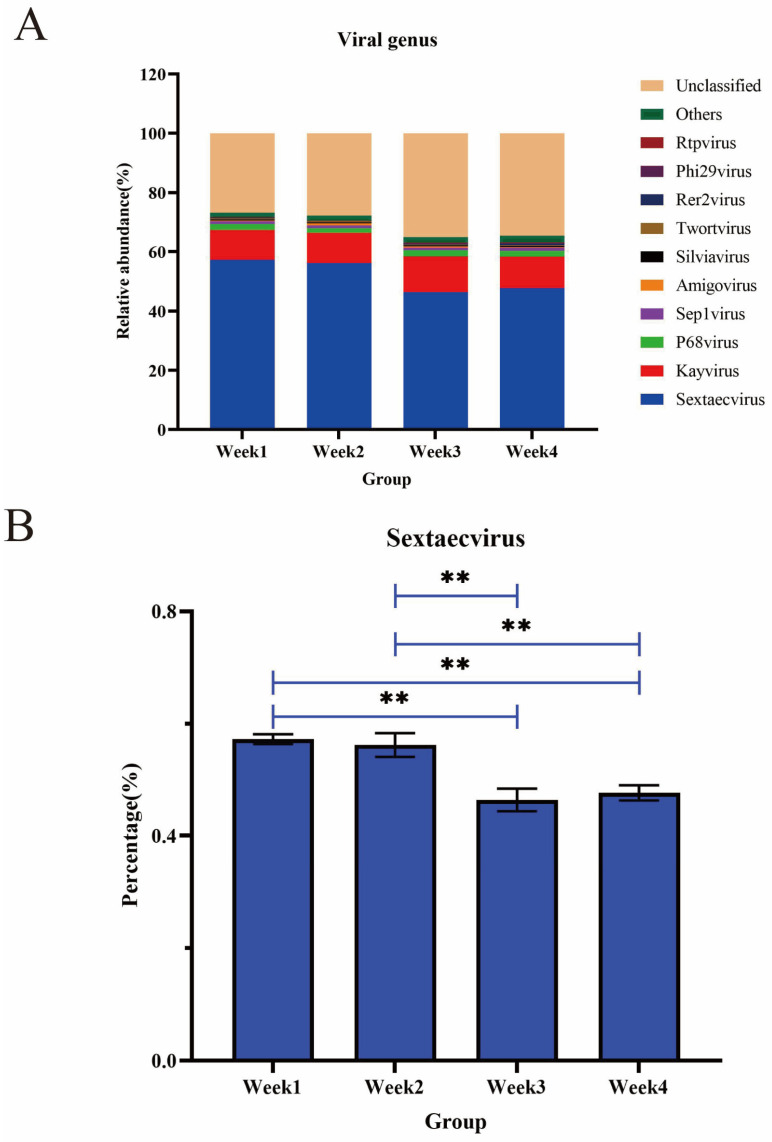
(**A**) The top 10 most abundant viral genera in the PM2.5 samples over four weeks. (**B**) The relative abundance changes in Sextaecvirus during four weeks. ** *p* < 0.01.

At the family and genus levels (the phylum-to-order taxa could not be assigned), 29 families and 188 genera were detected in the PM2.5 samples (Supplementary Table S2). The dominant viruses at the family level were *Siphoviridae*, *Myoviridae*, and *Podoviridae*, with average proportions of 67.00%, 25.21%, and 3.28%, respectively, while the ratio of other viruses was less than 1%. See Supplementary Table S4 for the specific types and percentages of families. The viral communities in all samples were mainly composed of *Sextaecvirus* (51.88%), *Kayvirus* (10.75%), and *P68virus* (1.95%) at the genus level (Figure 5A). Furthermore, a significant decrease in the relative abundance of *Sextaecvirus* was observed in all groups (Figure 5B). A high prevalence of *Sextaecvirus* was observed among the genera found, representing almost 50% of all viruses (Figure 5A). The alpha diversity index in viral communities was further analyzed (Supplementary Figure S3). No significant differences were observed in the ACE or Chao1 indexes among viruses. However, diversity variance was significantly higher in viral communities compared with other groups (*p* < 0.05). Furthermore, based on the Bray–Curtis distance, a dimensionality reduction analysis, the PCoA plots demonstrated that the PM2.5 samples were close to each other in viral structure (Supplementary Figure S4).

In our study, we also found eight kinds of avian viruses, namely Fowl aviadovirus E (948 strains), Fowl aviadovirus D (195 strains), Avian leukosis virus (99 strains), Avian endogenous retrovirus EAV-HP (64 strains), Avian dependent parvovirus 1 (29 strains), Fowl adenovus (23 strains), Fowl aviadovirus B (7 strains), and Avian sarcoma virus (2 strains). Four human viruses were also found in the PM2.5 samples, including Human mastadovirus C (263 strains), Human gut gokushovirus (37 strains), Human endogenous retrovirus K (28 strains), and Human endogenous retrovirus W (14 strains). Fowl aviadenovirus was the most abundant virus of poultry origin, among which avian adenovirus group I is the most harmful to chicks aged 3–5 weeks. The chicks infected mainly show inclusion body hepatitis, hepatitis–hydropericardium syndrome, and monogastric erosion symptoms [46]. During the sample collection process, there were no apparent symptoms of disease in the chickens, which may be due to the low amount of virus in the microbial aerosol of the chicken house or the strong resistance of the chickens themselves.

### 3.6. Diversity and Abundance of Archaea

The discovery of archaea has dramatically improved our understanding of biological adaptability under extreme conditions. Many kinds of archaea have been found to participate in the global biogeochemistry cycle. In this study, *Euryarchaeota*, which is traditionally called *Methanogen*, contained the most abundant groups. At the genus level, the relative abundance values of *Metanobrevibate* and *Metanocorpusculum* were the highest, at 45.48% and 22.77%, respectively (Supplementary Figure S5). These both belong to *Methanogen* and come from *Methanobacteria* and *Methanomicrobiales*, respectively. It was reported that the majority of *Methanogen* in the animal gastrointestinal tract was *Methanobrevibacter*, and that they were all hydrogen-trophic [47,48]. This report is similar to the results of this study. Methanogenic contributions towards pathogenicity have received much less attention than their bacterial counterparts. They have been found to be associated with a variety of diseases, such as abscess [49], periodontal disease [50], and colitis [51]. Therefore, as a part of the gut microbiota, archaea have both beneficial and harmful effects on health, and further research is still needed to confirm their role.

### 3.7. Functional Profile of the DNA Metagenome

**Figure 6:**
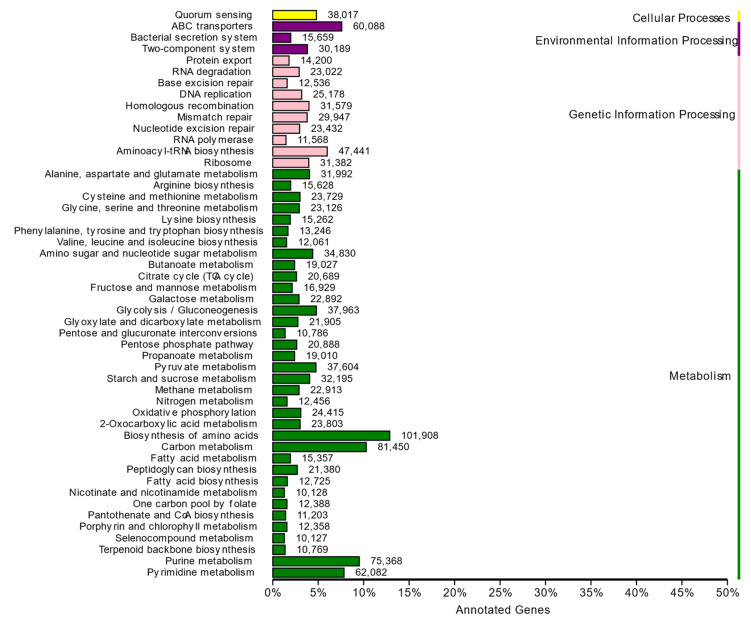
Analysis of predicted functions at KEGG level 2 for microbiota from the PM2.5 samples.

To obtain additional information to assess the functional potential associated with the microbial community, a set of PM2.5 DNA was applied in databases such as KEGG, CAZy, and VFDB. The protein sequences of non-redundant genes were compared by Blast to predict the functional distribution of microbial communities in the PM2.5 samples. KEGG results showed that the metabolic pathway was the most abundant pathway among the four major prediction pathways found, and more than 35% of genes were involved in metabolism. At KEGG level 2, global and overview maps were the most representative KEGG functional categories in metabolic pathways, followed by carbohydrate metabolism and amino acid metabolism (Figure 6). At KEGG level 3, 172 pathways were found. Among them, nine pathways belonged to global and overview maps, 15 to carbohydrate metabolism and 14 to amino acid metabolism.

CAZY carbohydrate-active enzymes have the ability to synthesize and degrade carbohydrates. CAZY carbohydrate-active enzymes have six functional categories: glycoside hydrolases (GHs), glycosyltransferases (GTs), polysaccharide lyases (PLs), carbohydrate esterases (CEs), auxiliary activities (AAs), and carbohydrate binding domains (CBMs). The relative content of functional genes for glycoside hydrolases (GHs) accounted for 42.7% of these, and the relative contents of functional genes for glycosyltransferases (GTs) and carbohydrate esterases (CEs) accounted for 26.3% and 14.7%, respectively (Supplementary Figure S6). Carbohydrate-active enzymes break down complex carbohydrates into components that the intestinal epithelium can absorb, and also provide an important pathway for microorganisms to participate in physiological and pathological processes in the body. Therefore, corresponding changes in carbohydrate-active enzymes can reflect the state of microorganisms and serve as indicators for studying microbial function.

After analyzing our sequencing data, we detected 355 virulence factors, likely the main reasons for the clinical symptoms in poultry infected with bacteria. The relative content of the annotated virulence factors was less than 1%; however, we also found that the top five virulence factors produced a high risk (Table 2). These were FbpABC (0.48%), HitABC (0.36%), colibactin (0.27%), acinetobactin (0.22%) and capsule (0.22%). The functions of FbpABC, HitABC, and acinetobactin are related to iron uptake, which indicates that the iron transport system may be an essential factor in reducing the content of microbial aerosols. Colibactin, from *Klebsiella* pneumonia, is the most dangerous virulence factor in the top five, because it has genetic toxicity and can induce DNA damage. Capsule comes from *Enterococcus faecalis*. It can escape host immunity by combining with C3 detected on the surface of fecal *Escherichia coli*. Virulence factor genes (VFG) carried by pathogens are considered the root cause of disease because they can encode VF, cause its pathogenic host to invade humans and/or animals, and then induce disease [52,53]. Therefore, pathogens containing VFG in the microbial aerosol are a huge hidden danger to environmental safety and a potential threat to animal and even human health.

## 4. Conclusions

Multiple pathogenic microorganisms were detected in the chicken house environment and could pose potential pathogenic risks. Metagenomic sequencing addressed the composition of PM2.5 and investigated changes in bacterial, fungal, and viral composition and the concentration of PM2.5 during a month of growth stages. In four weeks, the relative abundance of the two phyla from fungi had changed significantly. In this study, the composition of the virus was a significant result. The dominant viruses at the family level were *Siphoviridae, Myoviridae*, and *Podoviridae*, at average proportions of 67.00%, 25.21%, and 3.28%, respectively. In addition, 1338 strains of eight avian viruses were found. The results of functional prediction showed that there were more than 35% of genes that may be involved in metabolism. Metagenomic results comprehensively revealed the characteristics of microorganisms in the environment of the chicken house to better protect the environments of chicken houses and the health of chickens. Given the rapid development of animal husbandry, it is necessary to strictly regulate environmental microorganisms for animal disease prevention and control in animal sheds, in order to improve the welfare and production efficiency of these animals.

## Figures and Tables

**Table 1:** Over four weeks, the relative abundance and number of species of 10 common opportunistic pathogens.

Opportunistic Pathogens	Relative Abundance	Number of Species
*Staphylococcus*	15.999%	138
*Clostridium*	2.486%	456
*Enterococcus*	0.497%	80
*Escherichia*	0.294%	31
*Campylobacter*	0.158%	176
*Streptococcus*	0.097%	137
*Acinetobacter*	0.090%	60
*Mycobacterium*	0.024%	207
*Listeria*	0.019%	23
*Salmonella*	0.014%	4

**Table 2:** The function and abundance of the top five virulence factors.

Virulence Factor ID	VF’s Name	Species Name	VF’s Function	Abundance
VF0272	FbpABC	*Neisseria meningitidis*	Encodes a periplasmic-binding protein-dependent iron transport system necessary for the utilization of iron bound to transferrin or iron chelates; FbpA is the periplasmic Fe3+ binding protein	0.48%
VF0268	HitABC	*Haemophilus influenzae*	The HitABC(fbpABC) operon encodes a periplasmic-binding protein-dependent iron transport system necessary for the utilization of iron bound to transferrin or iron chelates	0.36%
VF0573	Colibactin	*Klebsiella pneumonia*	Inducing DNA damage	0.27%
VF0467	Acinetobactin	*Acinetobacter baumannii*	High-affinity catechol-hydroxamate siderophore competing with host cells for iron	0.22%
VF0361	Capsule	*Enterococcus faecalis*	Contributes to host immune evasion	0.22%

## Data Availability

Data are contained within the article and Supplementary Materials.

## Supplementary Materials

The following supporting information can be downloaded at: https://www.mdpi.com/article/10.3390/ani14010055/s1, Table S1: Evaluation statistics of sequencing data of 12 PM2.5 samples; Table S2: Number of microorganisms detected at each classification level; Table S3:Comparison of alpha diversity index for fungi in PM2.5 samples (n = 12); Table S4: Virus types and proportion at the family level; Figure S1: Alpha diversity based on species abundance at the genus level; Figure S2: Principal coordinate analysis plot generated using abundance at different taxonomic levels based on Bray–Curtis dissimilarities. Each plot represents a sample; Figure S3: Alpha diversity is based on species abundance at the genus level; Figure S4: PCoA plot generated using abundance at different taxonomic levels based on Bray–Curtis dissimilarities; Figure S5: The top 10 most abundant Archaea in PM2.5 samples; Figure S6: Taxonomy and relative abundances of the classifiable carbohydrate-active enzyme identified in the microbiota of PM2.5 during four weeks.
