# Supplementary material for: Exploring the Microbial Community Structure in the Chicken House Environment by Metagenomic Analysis
Source: Animals (Basel). 2023 Dec 22;14(1):55. doi: 10.3390/ani14010055 (PMC10778276; doi:10.3390/ani14010055)
Supplement: Supplementary file 1 [file animals-14-00055-s001.zip › animals-2741785-supplementary.pdf]

Supplementary materials

Exploring the Microbial Community Structure in the Chicken House Environment by Metagenomic Analysis

Cheng Lou[a] , Zhuo Chen[a] , Yu Bai[a], Tongjie Chai[b], Yuling Guan[a], Bo Wu[a,*]

[a] *Guangdong Provincial Key Laboratory of Animal Molecular Design and Precise Breeding, School of Life Science and Engineering, Foshan University, Foshan, China 528225*

[b] *College of Animal Science and Technology, Shandong Agricultural University, Tai'an 271000, China.*

[*] **Correspondence:** wubo@fosu.edu.cn**(B.W)**

Supplementary Table S1: Evaluation statistics of sequencing data of 12 PM2.5 samples.

Group	Sample ID	Raw data base(bp)	Clean data base(bp)	Number of Reads	GC(%)	Q20(%)	Q30(%)
Week1	W1M1	8152873112	8092464193	27027099	48.83	98.6	95.79
Week1	W1M2	6730284254	6667500725	22264075	48.34	98.51	95.59
Week1	W1M3	6217374690	6164374444	20589878	48.82	98.51	95.58
Week2	W2M1	9388987116	9296881735	31039190	48.54	98.53	95.62
Week2	W2M2	7678724900	7582268150	25312479	49.63	98.32	95.13
Week2	W2M3	8764339944	8680299691	28996033	49.05	98.48	95.51
Week3	W3M1	10570961438	10450221463	34906464	48.89	98.49	95.59
Week3	W3M2	7936186646	7805794828	26078312	48.83	97.72	93.67
Week3	W3M3	6887104106	6825073894	22809347	50.25	98.48	95.54
Week4	W4M1	7480615758	7414397453	24764618	49.01	98.45	95.41
Week4	W4M2	6622623486	6561317329	21913112	49.48	98.43	95.36
Week4	W4M3	7838815332	7757828554	25900877	48.23	98.32	95.03
Total		94268890782	93298422459	311601484	587.90	1180.84	1143.82
AVER		7855740898.50	7774868538.25	25966790.33	48.99	98.40	95.32

Note: Q20(%): Percentage of the base with a mass value greater than or equal to 20 in total base; Q30(%): Percentage of the base with a mass value greater than or equal to 30 in entire base; GC(%): GC content of a sample, namely the percentage of G and C type bases in total bases.

Supplementary Table S2:Number of microorganisms detected at each classification level.

Sources	Percentage	Phylum	Class	Order	Family	Genus	Species
Bacteria	89.80%	145	94	194	448	2377	19374
Fungi	1.08%	9	39	92	212	390	744
Virus	2.06%	1	1	4	29	188	1198
Archaea	0.49%	15	16	27	42	116	508
Sum	93.43%	170	150	317	731	3,071	21,824

Supplementary Table S3:Comparison of alpha diversity index for fungi in PM2.5 samples (n=12).

Group	Number of samples	Shannon[a]	Simpson[a]	ACE[a]	Chao1[a]
Week1	3	1.315±0.077	0.611±0.036	200.736±8.886	200.037±9.297
Week2	3	1.408±0.114	0.610±0.013	221.237±54.965	221.083±55.127
Week3	3	1.530±0.145	0.623±0.055	167.222±25.667	167.143±25.729
Week4	3	1.314±0.040	0.491±0.014	146.091±10.064	145.549±10.038
F		2.997	9.877	3.508	3.491
P		0.095	0.005	0.069	0.07

Note: [a] All data shown were $\overline{X}\pm S$ (mean±standard deviation). The *P* value was obtained with one-way ANOVA

Supplementary Table S4:Virus types and proportion at the family level.

Virus Classification (Family)	Percentage（%）
Siphoviridae	66.9965%
Myoviridae	25.2145%
Podoviridae	3.2807%
Adenoviridae	0.1258%
Herpesviridae	0.1223%
Retroviridae	0.0815%
Mimiviridae	0.0400%
Microviridae	0.0397%
Parvoviridae	0.0146%

Totiviridae	0.0074%
Ascoviridae	0.0064%
Caulimoviridae	0.0058%
Poxviridae	0.0051%
Phycodnaviridae	0.0049%
Circoviridae	0.0041%
Polydnaviridae	0.0031%
Baculoviridae	0.0028%
Pithoviridae	0.0022%
Nudiviridae	0.0020%
Alloherpesviridae	0.0017%
Iridoviridae	0.0016%
Marseilleviridae	0.0014%
Ackermannviridae	0.0011%
Smacoviridae	0.0010%
Chuviridae	0.0004%
Metaviridae	0.0003%
Geminiviridae	0.0003%
Flaviviridae	0.0002%
Genomoviridae	0.0002%
Unclassified	4.0325%

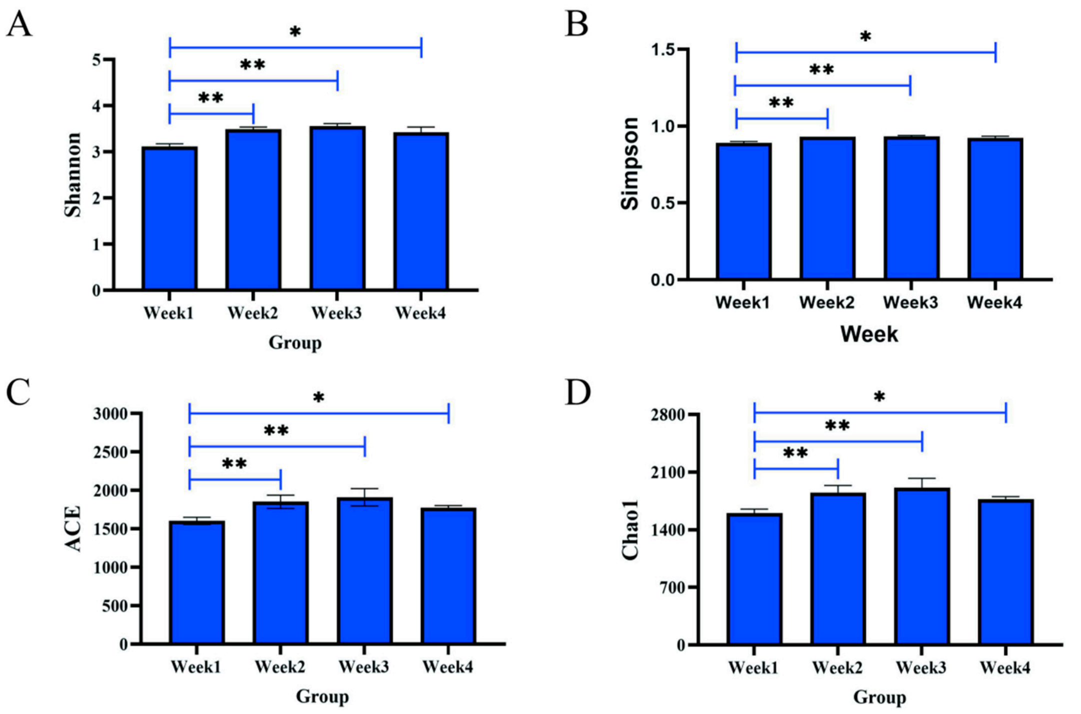

Supplementary Figure S1: Alpha diversity based on species abundance at the genus level.

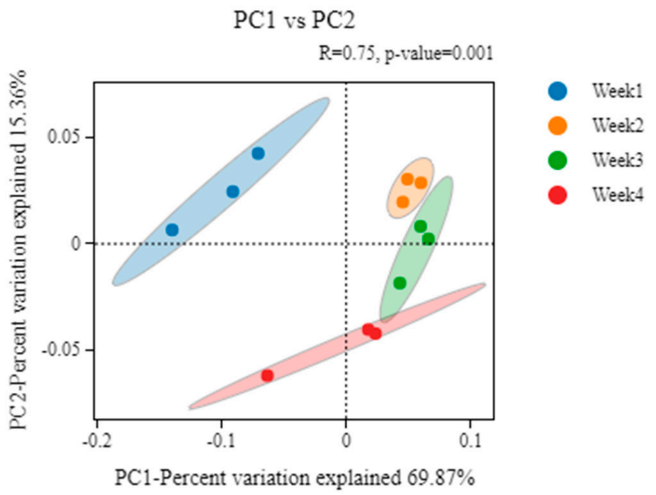

Supplementary Figure S2: Principal coordinate analysis plot generated using abundance at different taxonomic levels based on Bray - Curtis dissimilarities. Each plot represents a sample.

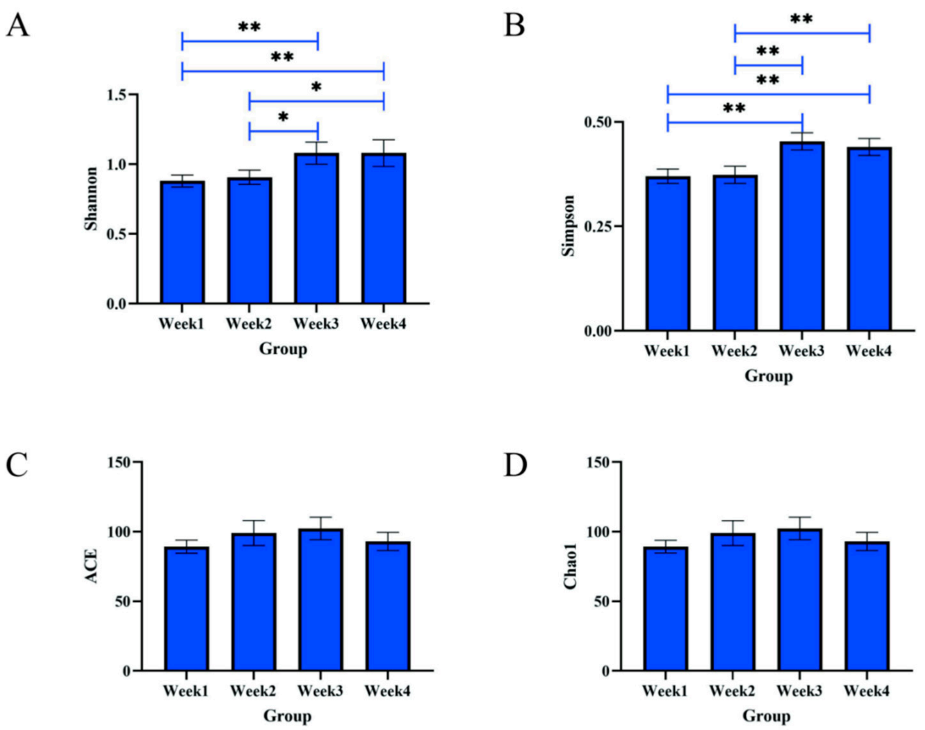

Supplementary Figure S3: Alpha diversity is based on species abundance at the genus level.

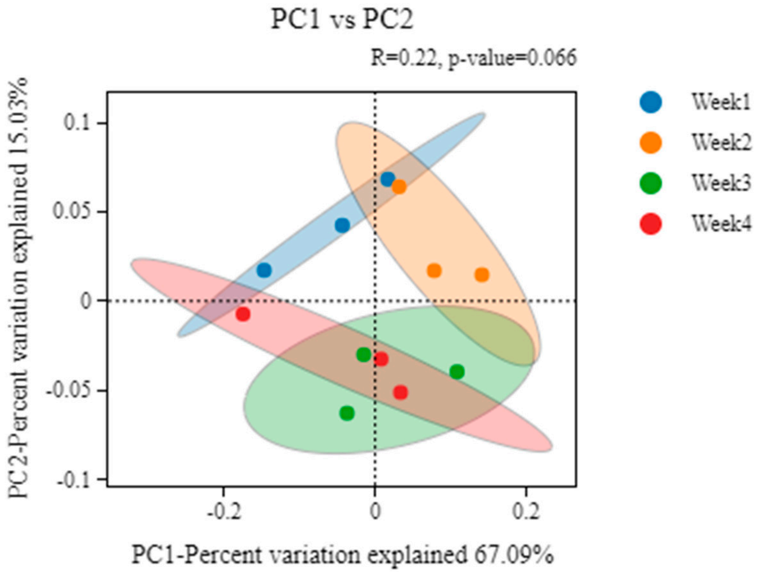

Supplementary Figure S4: PCoA plot generated using abundance at different taxonomic levels based on Bray–Curtis dissimilarities.

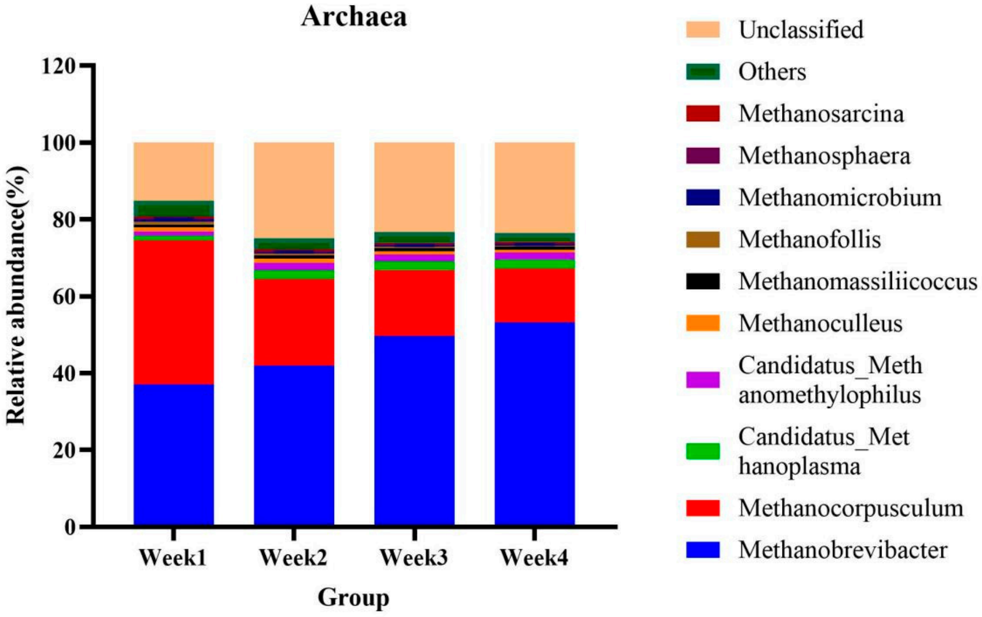

Supplementary Figure S5: The top 10 most abundant Archaea in PM2.5 samples.

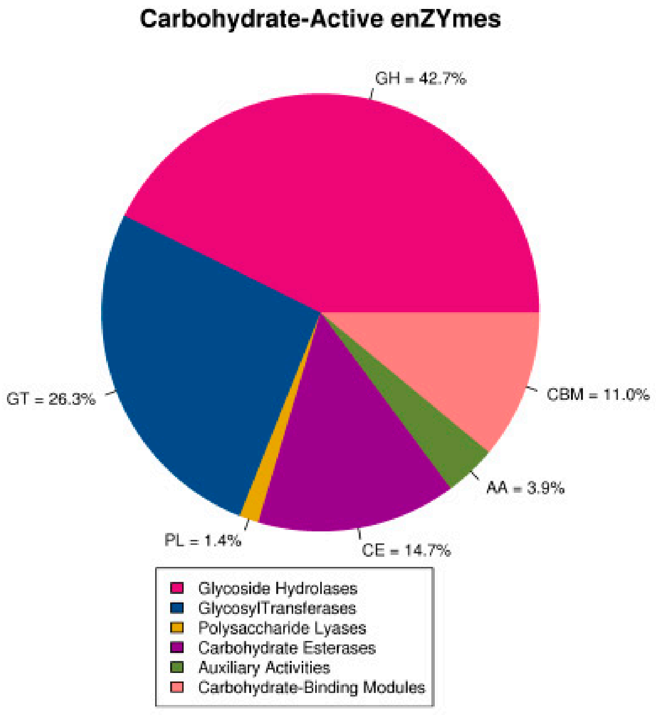

Supplementary Figure S6: Taxonomy and relative abundances of the classifiable carbohydrate-active enzyme identified in the microbiota of PM2.5 during four weeks.
